# Synthesis, optical properties and crystal structure of (*E*,*E*)-1,3-(3,4:9,10-dibenzododeca-1,11-diene-5,7-diyne-1,12-di­yl)benzene

**DOI:** 10.1107/S2056989023006187

**Published:** 2023-07-28

**Authors:** Hikaru Watanabe, Takuma Sato, Michiki Sumita, Mei Shiroyama, Daichi Sugawara, Tomoki Tokuyama, Yasuhiro Okuda, Kan Wakamatsu, Haruo Akashi, Akihiro Orita

**Affiliations:** a Department of Applied Chemistry, Okayama University of Science, 1-1 Ridai-cho, Kita-ku, Okayama 700-0005, Japan; b Department of Chemistry, Okayama University of Science, 1-1 Ridai-cho, Kita-ku, Okayama 700-0005, Japan; c Research Institute of Frontier Science and Technology, Okayama University of Science, 1-1 Ridai-cho, Kita-ku, Okayama 700-0005, Japan; University of Kentucky, USA

**Keywords:** crystal structure, expanded π-system, de­hydro­benzannulene, reductive de­sulfonyl­ation

## Abstract

The de­hydro­benzannulene (*E*,*E*)-1,3-(3,4:9,10-dibenzododeca-1,11-diene-5,7-diyne-1,12-di­yl)benzene, C_26_H_16_, was successfully synthesized *via* photocatalyst-assisted stereoselective reductive de­sulfonyl­ation of 1,3-bis­{1-phenyl­sulfonyl-2-[2-(tri­methyl­silylethyn­yl)phen­yl]ethen­yl}benzene, C_44_H_42_O_4_S_2_Si_2_, and subsequent desilylative cyclization of the resulting (*E*,*E*)-bis-silyl-protected dienyne, C_32_H_34_Si_2_. The structure of the de­hydro­benzannulene thus obtained was confirmed by single-crystal X-ray analysis; three benzene rings are connected to one another by a 1,3-butadiynylene and a pair of ethenylene arrays. Although the π-system expanded efficiently in the de­hydro­benzannulene, it was observed that the butadiynylene and ethenylene arrays were strained, showing smaller [171.3 (2)–172.6 (2) °] and larger bond angles [122.5 (2)–131.9 (2)°] than the conventional bond angles, respectively. In CHCl_3_, the de­hydro­benzannulene showed the longest absorption band at 377 nm. When irradiated by UV light, it emitted fluorescence at 468 nm (Φ_F_ = 0.26) and 504 nm (Φ_F_ = 0.24) in CHCl_3_ and in the powdered state, respectively.

## Abstract 2

A de­hydro­benzannulene, (*E*,*E*)-1,3-(3,4:9,10-dibenzododeca-1,11-diene-5,7-diyne-1,12-di­yl)benzene, was stereoselectively synthesized, and its crystal structure and UV-Vis absorption and photoluminescence optical properties were determined.

## 1. Chemical context

De­hydro­benzannulenes (DBAs) attract intensive attention because they often show new functionality for π-expanded compounds, such as a novel π–π inter­action mode in fluoro­aryl­ene-DBA (Karki *et al.*, 2022 ▸), guest-dependent structure-transformative DBA inclusion crystals (Shigemitsu *et al.*, 2012 ▸), and a synthetic inter­mediate of [6.8]_3_cyclacene (Esser *et al.*, 2008 ▸). In the syntheses of DBAs, ethenylene and ethynylene arrays are often used to connect aromatic rings to one another. For example, 1,3-(3,4:9,10-dibenzododeca-1,11-diene-5,7-diyne-1,12-di­yl)benzene, C_26_H_16_, (**1**), is composed of three phenyl rings, a single butadienylene and a couple of ethenylene arrays. The synthesis of **1** was accomplished in 1985 (Ojima *et al.*, 1985 ▸). The synthetic route of **1** reported by Ojima was rather straightforward, and the desired de­hydro­benzannulene **1** were successfully obtained. However, while the formation of (*E*,*E*)-**1** was spectroscopically confirmed, X-ray single crystallographic analysis has not yet been performed because of a poor chemical yield of (*E*,*E*)-**1** in Ojima’s route. Recently we established an (*E*)-stereoselective synthesis of di­aryl­ethene *via* photocatalyst-assisted reductive de­sulfonyl­ation of the corresponding di­aryl­ethenyl sulfone under irradiation by visible light (Watanabe *et al.*, 2020 ▸, 2021 ▸). It was found out that this protocol could produce (*E*,*E*)-**1** efficiently in a pure form. This work reports the synthesis of the de­hydro­benzannulene (*E*,*E*)-**1** and its single-crystal X-ray structure together with *UV* absorption and photoluminescence optical properties of (*E*,*E*)-**1** in CHCl_3_ solution and in the solid state. 
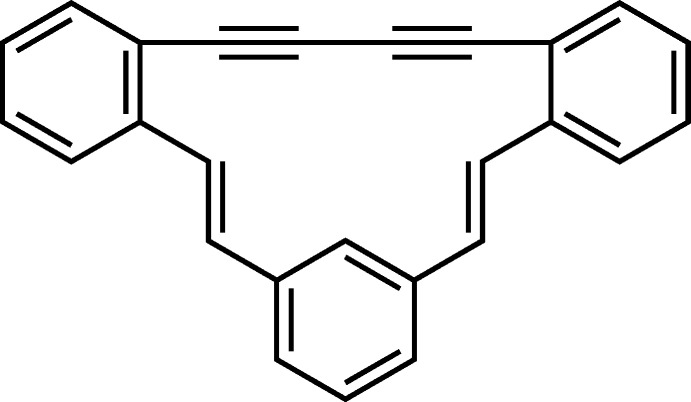


## 2. Structural commentary

**Figure 1:**
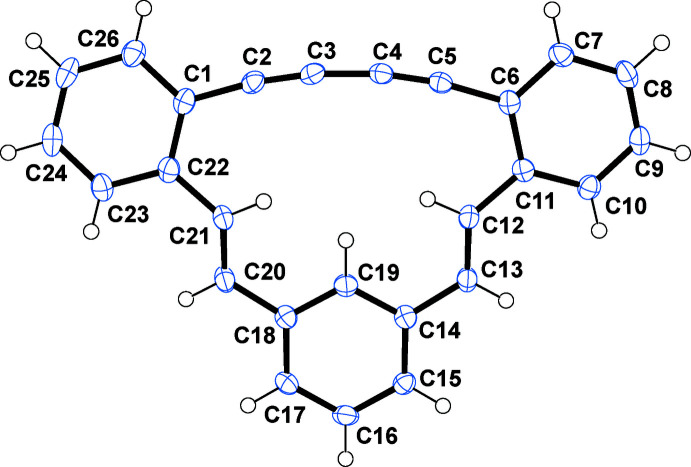
The mol­ecular structure of (*E*,*E*)-**1** with displacement ellipsoids drawn at the 50% probability level.

The core structure of (*E*,*E*)-**1** is a 15-membered ring in which three phenyl­ene rings are connected to one another by a 1,3-butadiynylene and a pair of (*E*)-ethenylene arrays (Fig. 1 ▸). Although the π-system in the 15-membered ring efficiently expands, there are slight twists observed in the π-systems between the (*E*)-ethenylene units and the connected phenyl­ene units: *e.g.* C19—C18—C20—C21 = −10.5 (4)° and C20—C21—C22—C23 = 13.1 (4)°. In the 1,3-butadiynylene array, triple bonds C2≡C3 [1.204 (3) Å] and C4≡C5 [1.199 (3) Å] are remarkably shorter than the central single bond C3—C4 [1.374 (3) Å] and terminal single bonds C1—C2 [1.434 (3) Å] and C5—C6 [1.439 (3) Å]. The former single bond, C3—C4, is shorter by 0.06 Å than the latter because of the strong π-conjugation between ethynylene moieties. In the pair of phenyl­enes, which are *ortho*-fused to the 15-membered ring (C1–C26 and C6–C11), the aromatic C—C junction bonds C1—C22 and C6—C11 are longer than the other phenyl­ene C—C bonds [1.414 (3) Å and 1.416 (3) Å *vs* 1.378 (4)–1.398 (3) Å] while in the *meta*-fused phenyl­ene ring (C14–C19), all the aromatic C—C bonds are essentially identical in length [1.387 (3)–1.393 (3) Å]. With respect to bond angles in the 15-membered ring, the *sp* carbons of the 1,3-butadiynylene moiety show somewhat smaller bond angles than the ideal value of 180°, whereas the *sp^2^* carbons in the pair of (*E*)-ethenylene arrays show bond angles larger than 120°. In the 1,3-butadiynylene moiety, the inner *sp* carbons (C3 and C4) have *ca* 0.6° smaller bond angles than the outer (C2 and C5): *e.g.* C2—C3—C4 = 172.0 (2)° *vs* C1—C2—C3 = 172.6 (2)°. In the (*E*)-ethenylene moieties, C12 and C21 show *ca* 9.0° larger bond angles than C13 and C20: *e.g.* C11—C12—C13 = 131.9 (2) ° *vs* C12—C13—C14 = 122.5 (2)°.

## 3. Supra­molecular features

**Figure 2:**
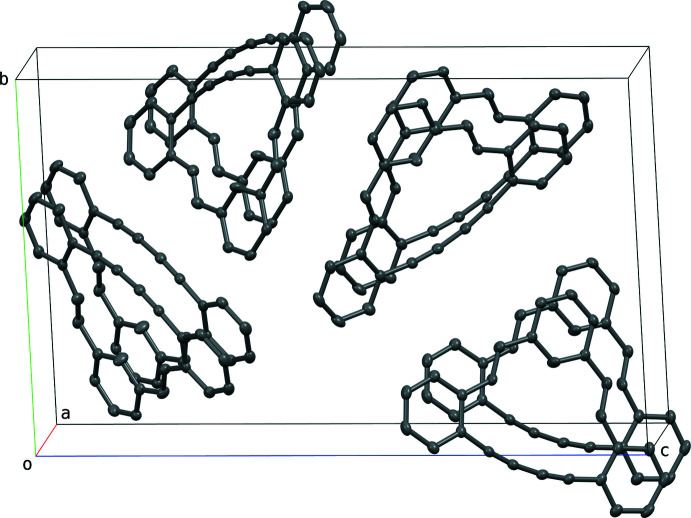
A partial packing plot of (*E*,*E*)-**1** viewed approximately down the crystallographic *a*-axis.

In the crystal, (*E*,*E*)-**1** mol­ecules form columnar structures that extend along the *a*-axis direction in which the inter­layer distance is 3.3639 (9) Å (calculated as the perpendicular distance from the mid-point of the 15-membered ring to the mean plane through the corresponding ring of an adjacent mol­ecule in the stack), indicating an efficient inter­molecular attractive inter­action through π–π stacking (Fig. 2 ▸). The columns in which the (*E*,*E*)-**1** mol­ecules are stacked are densely packed by van der Waals inter­actions.

## 4. Database survey

A search of the Cambridge Structural Database (version 5.43, November 2021 with updates to March 2022; Groom *et al.*, 2016 ▸) suggests the (*E*,*E*)-1,3-(3,4:9,10-dibenzododeca-1,11-diene-5,7-diyne-1,12-di­yl)benzene [(*E*,*E*)-**1**] structure is unprecedented, although the first synthesis of (*E*,*E*)-**1** and its spectroscopic assignment have been reported (Ojima *et al.*, 1985 ▸). The 1,4-diphenyl-1,3-butadiyne fragment in analogous DBA is, however, more common, with more than ten examples reported, including the close relative of tribenzo­tetra­yne DBA (refcode EKIMAM; Tobe *et al.*, 2003 ▸). The 1,3-bis­(phenyl­ethen­yl)benzene fragment in analogous DBA is also common, with more than ten examples reported including the close relative of meta­cyclo­phanetrienes (GOBJIR and GOGMAR; Esser *et al.*, 2008 ▸).

## 5. Synthesis and crystallization

**Figure 3:**
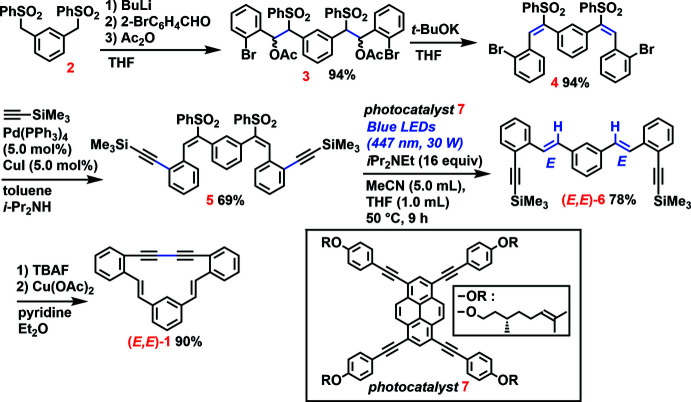
The synthetic route to (*E*,*E*)-**1**.

The de­hydro­benzannulene **1** was synthesized from **2** in five steps (Fig. 3 ▸). The starting di­sulfone **2** and π-expanded pyrene photocatalyst **7** were prepared according to the literature (Orita *et al.*, 2006 ▸; Watanabe *et al.*, 2021 ▸, respectively). A consecutive treatment of **2** with BuLi, 2-bromo­benzaldehyde, and acetic anhydride gave **3** in 94% yield as a diastereomeric mixture. The di­acetate **3** was successfully converted to **4** in a 94% yield by treatment with *t*-BuOK, and the resulting di­bromo­bis­(sulfonyl­ethen­yl)benzene **4** was transformed to **5** with a 69% yield *via* Sonogashira–Hagihara coupling with tri­methyl­silylethyne (Watanabe *et al.*, 2020 ▸). Subsequently our original photocatalyst-assisted reductive de­sulfonyl­ation was applied to bis­(1-phenyl­sulfonyl­ethen­yl)benzene **5** (Watanabe *et al.*, 2021 ▸). When blue light (447 nm, 30 W) was irradiated on a THF/MeCN solution of **5** in the presence of 5 mol% of pyrene photocatalyst **7** (2.5 mol% per sulfonyl­ethene moiety) and *i*-Pr_2_NEt as sacrificial reductant at 323 K for 9 h, the stereoselective reductive de­sulfonyl­ation proceeded smoothly to produce (*E*,*E*)-**6** in 78% yield. In contrast, during green-light irradiation (514 nm, 30 W), this de­sulfonyl­ation proceeded only sluggishly. When an ether/pyridine solution of **6** was treated with a THF solution of TBAF (tetra­butyl­ammonium fluoride), desilylation occurred rapidly to give terminal ethyne **8**. After the completion of the desilylation was confirmed by thin-layer chromatography (TLC) analysis, the final step, oxidative cyclization of the resulting terminal bis­yne **8**, was carried out in the presence of Cu(OAc)_2_ in air at 323 K for 3 h. The desired de­hydro­benzannulene **1** was obtained as yellow powder after column chromatography on silica gel. The spectroscopic data (^1^H NMR) were identical to that reported by Ojima *et al.* (1985 ▸).

**1,3-Bis(2-acet­oxy-2-(2-bromo­phen­yl)-1-phenyl­sulfonyl­eth­yl)benzene (3):** silica gel (hexa­ne/AcOEt, 6:4); a mixture of diastereomers; white powder; m.p 378–379 K; ^1^H NMR (CDCl_3_, 400 MHz): δ 1.86–2.33 (*m*, 6H), 4.27–5.10 (*m*, 2H), 6.57–7.22 (*m*, 8H), 7.26–7.87 (*m*, 14H); ^13^C{^1^H} NMR (CDCl_3_, 101 MHz): δ 20.76, 20.80, 20.9, 21.0, 21.1, 21.2, 70.3, 70.4, 71.0, 71.1, 73.2, 120.8, 127.3, 127.4, 127.6, 127.78, 127.81, 127.9, 128.00, 128.04, 128.2, 128.4, 128.5, 128.6, 128.7, 128.82, 128.85, 128.95, 128.98, 129.05, 129.08, 129.1, 129.16, 129.21, 129.7, 129.8, 129.9, 129.95, 130.04, 130.4, 130.6, 132.58, 132.63, 133.2, 133.3, 133.5, 133.6, 133.7, 133.8, 133.9, 134.0, 135.87, 135.94, 136.0, 136.3, 137.9, 138.9, 139.2, 168.9, 168.97, 169.02, 169.1. HRMS (MALDI–TOF): *m*/*z* [*M* + Na]^+^ calculated for C_38_H_32_NaO_8_S_2_ 860.9803; found: 860.9782.

**(** ***E,E*** **)-1,3-Bis(2-(2-bromo­phen­yl)-1-phenyl­sulfonyl­ethen­yl)benzene (4):** silica gel (hexa­ne/AcOEt, 6:4); white powder; m.p 434–435 K; ^1^H NMR (CDCl_3_, 400 MHz): δ 6.58–6.60 (*m*, 2H), 6.83–6.85 (*m*, 2H), 6.94–7.00 (*m*, 3H), 7.02 (*t*, 1H, *J* = 1.6 Hz), 7.10–7.14 (*m*, 2H), 7.37–7.41 (*m*, 4H), 7.52–7.60 (*m*, 8H), 8.16 (s, 2H); ^13^C{^1^H} NMR (CDCl_3_, 101 MHz): δ 125.4, 126.9, 128.4, 128.8, 129.0, 130.5, 130.7, 130.8, 131.6, 133.0, 133.2, 133.4, 133.5, 138.4, 138.5, 142.8. HRMS (MALDI–TOF): *m*/*z* [*M* + Na]^+^ calculated for C_34_H_24_Br_2_NaO_4_S_2_ 740.9380; found: 740.9382.

**(** ***E,E*** **)-1,3-Bis(2-(2-(tri­methyl­silylethyn­yl)phen­yl)-1-phenyl­sulfonyl­ethen­yl)benzene (5):** silica gel (hexa­ne/EtOAc, 8:2); white powder; m.p 444–445 K; ^1^H NMR (CDCl_3_, 400 MHz): δ 0.37 (*s*, 18H), 6.64 (*d*, 2H, *J* = 7.6 Hz), 6.92–7.00 (*m*, 5H), 7.06 (*t*, 1H, *J* = 8.0 Hz), 7.19–7.23 (*m*, 2H), 7.35 (*t*, 4H, *J* = 7.8 Hz), 7.48–7.55 (m, 8H), 8.50 (s, 2H); ^13^C{^1^H} NMR (CDCl_3_, 101 MHz): δ 0.09, 102.2, 102.3, 125.5, 127.9, 128.5, 128.8, 128.9, 129.1, 129.5, 131.8, 131.9, 132.8, 133.3, 135.0, 137.6, 139.0, 141.7 (One carbon signal appears to be missing due to overlap). HRMS (MALDI–TOF): *m*/*z* [*M* + Na]^+^ calculated for C_44_H_42_NaO_4_S_2_Si_2_ 777.1961; found: 777.1937.

*Synthetic procedure from **5** to (*E*,*E*)-**6***

To a round-bottomed flask charged with a magnetic stirrer bar were added ethenyl sulfone **5** (188.5 mg, 0.250 mmol), **7** (15.2 mg, 12.5 µmol), *i*-Pr_2_NEt (0.70 mL, 4.0 mmol), MeCN (2.5 mL) and THF (0.5 mL). The flask was placed in a glass water-bath surrounded by blue strip lighting, and blue light was irradiated to the mixture for 9 h. During the photoreaction, the bath temperature was kept at 323–328 K because of heat radiation from the photoreactor. The mixture was evaporated, and the crude product was subjected to flash chromatography (hexa­ne/CH_2_Cl_2_, 9:1) to afford the desired (*E*,*E*)-**6** (92.6 mg, 0.195 mmol, 78% yield).

**(** ***E,E*** **)-1,3-Bis{2-[2-(tri­methyl­silylethyn­yl)phen­yl]ethen­yl}benzene [(** ***E,E*** **)-6]:** yellow powder; m.p 380–381 K; ^1^H NMR (CDCl_3_, 400 MHz): δ 0.32 (*s*, 18H), 7.17–7.22 (*m*, 4H), 7.31–7.35 (*m*, 2H), 7.39 (*t*, 1H, *J* = 7.7 Hz), 7.47–7.50 (*m*, 4H), 7.66 (*s*, 1H), 7.68 (*d*, 2H, *J* = 7.7 Hz), 7.73 (*d*, 2H, *J* = 16.4 Hz); ^13^C{^1^H} NMR (CDCl_3_, 101 MHz): δ 0.23, 99.9, 103.7, 124.6, 125.85, 125.91, 127.32, 127.35, 128.9, 129.2, 130.0, 132.9, 138.0, 139.2 (One carbon signal appears to be missing due to overlap). HRMS (MALDI–TOF): *m*/*z* [*M*]^+^ calculated for C_32_H_34_Si_2_ 474.2199; found: 474.2238.

*Synthetic procedure from (*E*,*E*)-**6** to (*E*,*E*)-**1***

To an ether (3.3 mL) and pyridine (1.1 mL) solution of **6** (47.5 mg, 0.10 mmol) was added a THF solution of TBAF (1.0 *M*, 0.22 mL, 0.22 mmol) at 273 K, and the mixture was stirred at rt for 3 h. The mixture was added to an ether (3.3 mL) and pyridine (1.1 mL) solution of Cu(OAc)_2_ (228 mg, 1.3 mmol), and the mixture was stirred at 323 K for 3 h. The mixture was poured into sat. NH_4_Cl aqueous solution and AcOEt, and the organic and aqueous layers were separated. The aqueous layer was extracted with AcOEt, and the combined organic layer was washed with water and brine. After drying over MgSO_4_, the solution was evaporated. The residue was subjected to column chromatography on silica gel (hexa­ne/CH_2_Cl_2_, 9:1) to provide **1** (29.6 mg, 0.090 mmol, 90% yield).

**(** ***E,E*** **)-1,3-(3,4:9,10-dibenzododeca-1,11-diene-5,7-diyne-1,12-di­yl)benzene ((** ***E,E*** **)-1):** yellow powder; m.p. 520–521 K; ^1^H NMR (CDCl_3_, 400 MHz): δ 7.14 (*d*, 2H, *J* = 16.4 Hz), 7.20–7.24 (*m*, 4H), 7.30 (*dd*, 1H, *J* = 8.2, 6.4 Hz), 7.36–7.40 (*m*, 2H), 7.42–7.44 (*m*, 2H), 7.71 (*d*, 2H, *J* = 8.2 Hz), 8.23 (*d*, 2H, *J* = 16.4 Hz), 8.65 (s, 1H); ^13^C{^1^H} NMR (CDCl_3_, 101 MHz): δ 81.1, 84.9, 121.9, 124.4, 125.0, 127.0, 127.5, 128.87, 128.92, 129.6, 130.7, 130.8, 139.2, 141.7.

The crystal of (*E*,*E*)-**1** used for X-ray diffraction was obtained from slow evaporation of a CH_2_Cl_2_/hexane solution.

## 6. Optical properties

**Figure 4:**
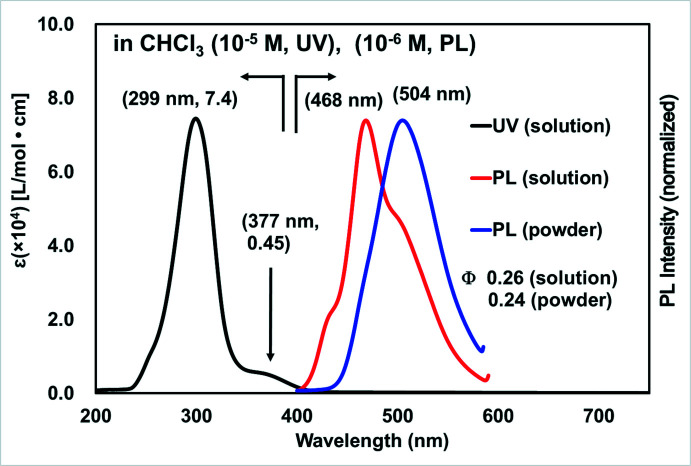
UV–Vis absorption and photoluminescence spectra of (*E*,*E*)-**1**.

**Figure 5:**
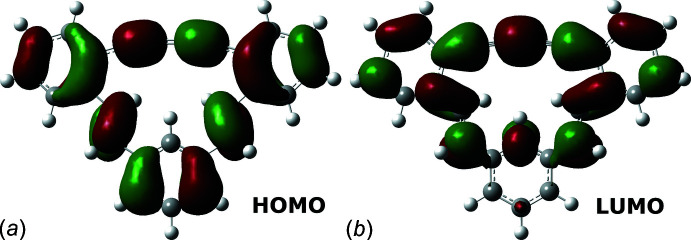
Graphical representation of frontier orbitals (*a*) HOMO and (*b*) LUMO of (*E*,*E*)-**1**.

To evaluate the electronic effects of the mol­ecular structure of (*E*,*E*)-**1** on its optical properties, UV–Vis absorption and photoluminescence spectra were recorded in CHCl_3_ (Fig. 4 ▸). In the UV–Vis absorption spectrum, (*E*,*E*)-**1** showed the longest and the maximum absorption bands at 377 nm (*ɛ* 0.45 × 10^4^ L mol^−1^ cm) and 299 nm (*ɛ* 7.4 × 10^4^ L mol^−1^ cm), respectively. The former absorption band was assignable to the HOMO–LUMO transition of (*E*,*E*)-**1** by DFT calculations performed at the B3LYP/6-31G(d) level of theory; 419 nm and *f* = 0.0415 were obtained as the first excitation energy and oscillator strength after calibration by multiplying by 0.96. The DFT calculations also revealed that the HOMO and LUMO of (*E*,*E*)-**1** expanded in the whole mol­ecule (Fig. 5 ▸). When UV light was irradiated to the CHCl_3_ solution of (*E*,*E*)-**1** and in the powdered state, blue and greenish blue-colored emissions were recorded at 468 nm (*Φ*_F_ 0.26) and 504 nm (*Φ*_F_ 0.24), respectively (Fig. 4 ▸).

## 7. Refinement

Crystal data, data collection and structure refinement details are summarized in Table 1 ▸. All H atoms were refined using a riding model with *d*(C—H) = 0.93 Å, *U*_iso_(H) = 1.2*U*_eq_(C) for aromatic H, 1.00 Å, *U*_iso_(H) = 1.2*U*_eq_(C) for CH, 0.98 Å.

## Supplementary Material

Crystal structure: contains datablock(s) I. DOI: 10.1107/S2056989023006187/pk2689sup1.cif

Supporting information file. DOI: 10.1107/S2056989023006187/pk2689Isup3.cdx

Supporting information file. DOI: 10.1107/S2056989023006187/pk2689Isup4.cml

Structure factors: contains datablock(s) I. DOI: 10.1107/S2056989023006187/pk2689Isup2.hkl

CCDC reference: 2252157

Additional supporting information:  crystallographic information; 3D view; checkCIF report

## Figures and Tables

**Table 1:** Experimental details

Crystal data	
Chemical formula	C_26_H_16_
*M*_r_	328.39
Crystal system, space group	Orthorhombic, *P*2_1_2_1_2_1_
Temperature (K)	293
*a*, *b*, *c* (Å)	4.6034 (2), 15.1542 (7), 24.1754 (9)
*V* (Å^3^)	1686.50 (12)
*Z*	4
Radiation type	Mo *K*α
μ (mm^−1^)	0.07
Crystal size (mm)	0.3 × 0.1 × 0.02

Data collection	
Diffractometer	Rigaku VariMax with Saturn
Absorption correction	Multi-scan (*CrysAlis PRO*; Rigaku OD, 2019 ▸)
*T*_min_, *T*_max_	0.739, 1.000
No. of measured, independent and observed [*I* > 2σ(*I*)] reflections	32971, 5357, 4184
*R*_int_	0.067
(sin θ/λ)_max_ (Å^−1^)	0.737

Refinement	
*R*[*F* ^2^ > 2σ(*F* ^2^)], *wR*(*F* ^2^), *S*	0.058, 0.115, 1.03
No. of reflections	5357
No. of parameters	235
H-atom treatment	H-atom parameters constrained
Δρ_max_, Δρ_min_ (e Å^−3^)	0.25, −0.21
Absolute structure	Undetermined: Flack *x* obtained using 1343 quotients [(*I* ^+^)−(*I* ^−^)]/[(*I* ^+^)+(*I* ^−^)] (Parsons *et al.* (2013 ▸)
Absolute structure parameter	−0.4 (10)

Computer programs: *CrysAlis PRO* (Rigaku OD, 2019 ▸), *SHELXT* (Sheldrick, 2015*a* ▸), *SHELXL* (Sheldrick, 2015*b* ▸) and *OLEX2* (Dolomanov *et al.*, 2009 ▸).

## supplementary crystallographic information

### Crystal data

**Table:**

C_26_H_16_	*D*_x_ = 1.293 Mg m^−^^3^
*M**_r_* = 328.39	Mo *K*α radiation, λ = 0.71073 Å
Orthorhombic, *P*2_1_2_1_2_1_	Cell parameters from 10472 reflections
*a* = 4.6034 (2) Å	θ = 2.1–31.6°
*b* = 15.1542 (7) Å	µ = 0.07 mm^−^^1^
*c* = 24.1754 (9) Å	*T* = 293 K
*V* = 1686.50 (12) Å^3^	Needle, pale yellow
*Z* = 4	0.3 × 0.1 × 0.02 mm
*F*(000) = 688	

### Data collection

**Table:**

Rigaku VariMax with Saturn diffractometer	5357 independent reflections
Radiation source: fine-focus sealed X-ray tube, Enhance (Mo) X-ray Source	4184 reflections with *I* > 2σ(*I*)
Graphite monochromator	*R*_int_ = 0.067
ω scans	θ_max_ = 31.6°, θ_min_ = 2.7°
Absorption correction: multi-scan (CrysAlisPro; Rigaku OD, 2019)	*h* = −6→6
*T*_min_ = 0.739, *T*_max_ = 1.000	*k* = −22→22
32971 measured reflections	*l* = −35→35

### Refinement

**Table:**

Refinement on *F*^2^	Hydrogen site location: inferred from neighbouring sites
Least-squares matrix: full	H-atom parameters constrained
*R*[*F*^2^ > 2σ(*F*^2^)] = 0.058	*w* = 1/[σ^2^(*F*_o_^2^) + (0.0399*P*)^2^ + 0.5407*P*] where *P* = (*F*_o_^2^ + 2*F*_c_^2^)/3
*wR*(*F*^2^) = 0.115	(Δ/σ)_max_ < 0.001
*S* = 1.03	Δρ_max_ = 0.25 e Å^−^^3^
5357 reflections	Δρ_min_ = −0.21 e Å^−^^3^
235 parameters	Absolute structure: Flack *x* obtained using 1343 quotients [(*I*^+^)-(*I*^-^)]/[(*I*^+^)+(*I*^-^)] (Parsons *et al.* (2013)
0 restraints	Absolute structure parameter: −0.4 (10)
Primary atom site location: dual	

### Special details

**Table:**

Geometry. All esds (except the esd in the dihedral angle between two l.s. planes) are estimated using the full covariance matrix. The cell esds are taken into account individually in the estimation of esds in distances, angles and torsion angles; correlations between esds in cell parameters are only used when they are defined by crystal symmetry. An approximate (isotropic) treatment of cell esds is used for estimating esds involving l.s. planes.

### Fractional atomic coordinates and isotropic or equivalent isotropic displacement parameters (Å^2^)

**Table:**

	*x*	*y*	*z*	*U*_iso_*/*U*_eq_	
C1	0.2502 (5)	0.45575 (15)	0.56944 (9)	0.0216 (5)	
C2	0.4402 (5)	0.47355 (14)	0.61508 (9)	0.0222 (5)	
C3	0.5925 (5)	0.49842 (14)	0.65244 (9)	0.0227 (5)	
C4	0.7514 (5)	0.53835 (14)	0.69355 (9)	0.0222 (5)	
C5	0.8704 (5)	0.58321 (15)	0.72752 (9)	0.0209 (4)	
C6	0.9800 (5)	0.64594 (14)	0.76707 (8)	0.0204 (4)	
C7	1.1873 (5)	0.62079 (15)	0.80626 (9)	0.0245 (5)	
H7	1.262529	0.563841	0.805862	0.029*	
C8	1.2806 (6)	0.68078 (16)	0.84569 (9)	0.0274 (5)	
H8	1.417729	0.664064	0.871925	0.033*	
C9	1.1693 (5)	0.76560 (16)	0.84596 (9)	0.0259 (5)	
H9	1.230444	0.805469	0.872792	0.031*	
C10	0.9683 (5)	0.79169 (15)	0.80678 (9)	0.0234 (5)	
H10	0.898169	0.849244	0.807273	0.028*	
C11	0.8687 (5)	0.73307 (14)	0.76650 (8)	0.0202 (4)	
C12	0.6595 (5)	0.75690 (15)	0.72402 (9)	0.0242 (5)	
H12	0.618597	0.711742	0.699159	0.029*	
C13	0.5176 (5)	0.83044 (15)	0.71427 (9)	0.0248 (5)	
H13	0.550528	0.878785	0.737125	0.030*	
C14	0.3080 (5)	0.83928 (15)	0.66845 (9)	0.0219 (5)	
C15	0.1742 (5)	0.91902 (15)	0.65530 (9)	0.0217 (5)	
H15	0.218318	0.969579	0.675416	0.026*	
C16	−0.0247 (6)	0.92324 (15)	0.61229 (9)	0.0262 (5)	
H16	−0.114100	0.976703	0.604104	0.031*	
C17	−0.0926 (5)	0.84895 (15)	0.58126 (9)	0.0235 (5)	
H17	−0.226582	0.852844	0.552574	0.028*	
C18	0.0403 (5)	0.76861 (15)	0.59313 (8)	0.0215 (5)	
C19	0.2331 (6)	0.76584 (16)	0.63734 (10)	0.0306 (6)	
H19	0.316351	0.711884	0.646541	0.037*	
C20	−0.0222 (5)	0.68716 (16)	0.56204 (9)	0.0254 (5)	
H20	−0.174216	0.687321	0.536702	0.030*	
C21	0.1283 (6)	0.61437 (16)	0.56873 (9)	0.0290 (5)	
H21	0.285437	0.620009	0.592654	0.035*	
C22	0.0935 (5)	0.52624 (15)	0.54539 (9)	0.0228 (5)	
C23	−0.0897 (6)	0.50780 (16)	0.50078 (9)	0.0261 (5)	
H23	−0.194796	0.553344	0.484524	0.031*	
C24	−0.1173 (6)	0.42325 (17)	0.48049 (10)	0.0291 (5)	
H24	−0.238731	0.412385	0.450511	0.035*	
C25	0.0346 (6)	0.35453 (16)	0.50444 (10)	0.0311 (6)	
H25	0.013435	0.297496	0.490833	0.037*	
C26	0.2182 (6)	0.37044 (16)	0.54866 (10)	0.0270 (5)	
H26	0.320639	0.324050	0.564593	0.032*	

### Atomic displacement parameters (Å^2^)

**Table:**

	*U*^11^	*U*^22^	*U*^33^	*U*^12^	*U*^13^	*U*^23^
C1	0.0218 (11)	0.0244 (11)	0.0187 (10)	−0.0033 (9)	0.0055 (9)	−0.0038 (8)
C2	0.0271 (12)	0.0193 (10)	0.0203 (10)	0.0011 (9)	0.0039 (9)	−0.0021 (8)
C3	0.0274 (12)	0.0174 (10)	0.0232 (10)	0.0019 (9)	0.0039 (9)	−0.0002 (8)
C4	0.0247 (12)	0.0199 (10)	0.0220 (10)	0.0030 (9)	−0.0004 (9)	0.0031 (8)
C5	0.0226 (11)	0.0209 (10)	0.0192 (10)	0.0018 (9)	0.0004 (9)	0.0036 (8)
C6	0.0206 (11)	0.0241 (11)	0.0165 (9)	−0.0035 (9)	0.0015 (9)	0.0015 (8)
C7	0.0251 (12)	0.0250 (11)	0.0233 (11)	−0.0017 (10)	−0.0007 (9)	0.0060 (9)
C8	0.0257 (12)	0.0350 (13)	0.0213 (11)	−0.0047 (11)	−0.0069 (10)	0.0051 (10)
C9	0.0262 (12)	0.0318 (12)	0.0197 (10)	−0.0081 (10)	−0.0022 (9)	−0.0020 (9)
C10	0.0238 (12)	0.0234 (11)	0.0230 (10)	−0.0031 (9)	0.0012 (10)	−0.0005 (8)
C11	0.0205 (11)	0.0234 (11)	0.0167 (9)	−0.0018 (9)	0.0022 (9)	0.0018 (8)
C12	0.0294 (13)	0.0223 (11)	0.0209 (10)	−0.0027 (10)	−0.0046 (10)	−0.0024 (8)
C13	0.0249 (12)	0.0248 (11)	0.0246 (11)	0.0006 (10)	−0.0054 (9)	−0.0055 (9)
C14	0.0206 (11)	0.0246 (11)	0.0204 (10)	0.0011 (9)	0.0010 (9)	−0.0013 (8)
C15	0.0240 (12)	0.0199 (10)	0.0212 (11)	−0.0018 (9)	0.0024 (9)	−0.0004 (8)
C16	0.0337 (14)	0.0212 (11)	0.0237 (11)	0.0051 (11)	0.0000 (11)	0.0048 (9)
C17	0.0259 (12)	0.0269 (12)	0.0177 (10)	0.0017 (10)	−0.0009 (9)	0.0035 (8)
C18	0.0209 (11)	0.0248 (11)	0.0189 (10)	0.0013 (9)	0.0014 (9)	−0.0008 (8)
C19	0.0342 (14)	0.0233 (12)	0.0343 (13)	0.0107 (11)	−0.0111 (11)	−0.0060 (10)
C20	0.0241 (12)	0.0309 (12)	0.0211 (10)	0.0012 (10)	−0.0054 (9)	−0.0037 (9)
C21	0.0379 (15)	0.0264 (12)	0.0225 (11)	−0.0033 (11)	−0.0116 (11)	0.0004 (9)
C22	0.0258 (12)	0.0256 (11)	0.0170 (9)	−0.0047 (10)	0.0033 (9)	−0.0005 (8)
C23	0.0275 (12)	0.0319 (12)	0.0190 (10)	−0.0035 (10)	−0.0010 (9)	−0.0007 (9)
C24	0.0257 (13)	0.0398 (14)	0.0217 (11)	−0.0064 (11)	−0.0001 (10)	−0.0098 (10)
C25	0.0332 (14)	0.0285 (12)	0.0315 (12)	−0.0047 (11)	0.0021 (11)	−0.0139 (10)
C26	0.0274 (13)	0.0269 (12)	0.0267 (11)	0.0008 (10)	0.0021 (10)	−0.0064 (9)

### Geometric parameters (Å, º)

**Table:**

C1—C2	1.434 (3)	C14—C19	1.387 (3)
C1—C22	1.414 (3)	C15—H15	0.9300
C1—C26	1.395 (3)	C15—C16	1.387 (3)
C2—C3	1.204 (3)	C16—H16	0.9300
C3—C4	1.374 (3)	C16—C17	1.389 (3)
C4—C5	1.199 (3)	C17—H17	0.9300
C5—C6	1.439 (3)	C17—C18	1.392 (3)
C6—C7	1.398 (3)	C18—C19	1.390 (3)
C6—C11	1.416 (3)	C18—C20	1.474 (3)
C7—H7	0.9300	C19—H19	0.9300
C7—C8	1.386 (3)	C20—H20	0.9300
C8—H8	0.9300	C20—C21	1.313 (3)
C8—C9	1.384 (3)	C21—H21	0.9300
C9—H9	0.9300	C21—C22	1.459 (3)
C9—C10	1.382 (3)	C22—C23	1.397 (3)
C10—H10	0.9300	C23—H23	0.9300
C10—C11	1.396 (3)	C23—C24	1.378 (3)
C11—C12	1.454 (3)	C24—H24	0.9300
C12—H12	0.9300	C24—C25	1.382 (4)
C12—C13	1.313 (3)	C25—H25	0.9300
C13—H13	0.9300	C25—C26	1.384 (3)
C13—C14	1.475 (3)	C26—H26	0.9300
C14—C15	1.393 (3)		
			
C22—C1—C2	119.0 (2)	C16—C15—C14	120.2 (2)
C26—C1—C2	121.1 (2)	C16—C15—H15	119.9
C26—C1—C22	119.9 (2)	C15—C16—H16	119.5
C3—C2—C1	172.6 (2)	C15—C16—C17	121.1 (2)
C2—C3—C4	172.0 (2)	C17—C16—H16	119.5
C5—C4—C3	171.3 (2)	C16—C17—H17	120.0
C4—C5—C6	171.8 (2)	C16—C17—C18	119.9 (2)
C7—C6—C5	120.6 (2)	C18—C17—H17	120.0
C7—C6—C11	120.5 (2)	C17—C18—C20	122.8 (2)
C11—C6—C5	118.9 (2)	C19—C18—C17	117.7 (2)
C6—C7—H7	120.0	C19—C18—C20	119.5 (2)
C8—C7—C6	119.9 (2)	C14—C19—C18	123.5 (2)
C8—C7—H7	120.0	C14—C19—H19	118.3
C7—C8—H8	120.1	C18—C19—H19	118.3
C9—C8—C7	119.9 (2)	C18—C20—H20	118.7
C9—C8—H8	120.1	C21—C20—C18	122.5 (2)
C8—C9—H9	119.7	C21—C20—H20	118.7
C10—C9—C8	120.7 (2)	C20—C21—H21	114.2
C10—C9—H9	119.7	C20—C21—C22	131.6 (2)
C9—C10—H10	119.5	C22—C21—H21	114.2
C9—C10—C11	121.1 (2)	C1—C22—C21	118.5 (2)
C11—C10—H10	119.5	C23—C22—C1	118.3 (2)
C6—C11—C12	118.6 (2)	C23—C22—C21	123.2 (2)
C10—C11—C6	117.9 (2)	C22—C23—H23	119.5
C10—C11—C12	123.5 (2)	C24—C23—C22	121.1 (2)
C11—C12—H12	114.1	C24—C23—H23	119.5
C13—C12—C11	131.9 (2)	C23—C24—H24	119.8
C13—C12—H12	114.1	C23—C24—C25	120.3 (2)
C12—C13—H13	118.8	C25—C24—H24	119.8
C12—C13—C14	122.5 (2)	C24—C25—H25	119.9
C14—C13—H13	118.8	C24—C25—C26	120.1 (2)
C15—C14—C13	122.6 (2)	C26—C25—H25	119.9
C19—C14—C13	119.8 (2)	C1—C26—H26	119.9
C19—C14—C15	117.5 (2)	C25—C26—C1	120.3 (2)
C14—C15—H15	119.9	C25—C26—H26	119.9
